# Phylogenetic and phylodynamic analysis of respiratory syncytial virus strains circulating in children less than five years of age in Karachi-Pakistan

**DOI:** 10.1016/j.meegid.2024.105694

**Published:** 2024-11-26

**Authors:** Fatima Aziz, Nida Farooqui, Tanveer Abbas, Mahnoor Javaid, Wardah Rafaqat, Alnara Zhamalbekova, Syed Asad Ali, Syed Ali, Syed Hani Abid

**Affiliations:** a Department of Paediatrics and Child Health, Aga Khan University, Karachi, Pakistan; b Department of Microbiology, University of Karachi, Karachi, Pakistan; c Department of Biological and Biomedical Sciences, Aga Khan University, Karachi, Pakistan; d Medical College, Aga Khan University, Karachi, Pakistan; e School of Medicine, Nazarbayev University, Astana, Kazakhstan; f Department of Community Health Sciences, Aga Khan University, Karachi, Pakistan; g Department of Biomedical Sciences, School of Medicine, Nazarbayev University, Astana, Kazakhstan

**Keywords:** RSV, Genetic diversity, Phylogenetics, Phylodynamics, Molecular evolution

## Abstract

**Background:**

Respiratory syncytial virus (RSV) is one of the leading causes of infant morbidity and mortality worldwide, especially in Pakistan. To date, few studies have explored RSV epidemiology in different areas of Pakistan. However, none have performed comprehensive phylogenetic and phylodynamic analyses of RSV strains. This study presents a comprehensive genetic and phylodynamic analysis of RSV strains in children less than five years old in Karachi, Pakistan.

**Methods:**

This study used retrospectively collected nasopharyngeal (swab) samples from 155 children with qPCR-confirmed RSV infection. The samples were used to perform RSV genotyping using PCR employing RSV glycoprotein gene-specific primers. The RSVA and RSVB genotyping was performed using BLAST and Maximum-likelihood (ML) phylogenetic methods. Similarly, the relationship with other RSV strains was analyzed using ML phylogenetic cluster analysis. The RSVA and RSVB mean genetic diversity and coefficient of differentiation were calculated using MEGA7 software. Furthermore, the time to the most common recent ancestor (tMRCA) and effective population size of RSV genotypes A and B were estimated using a Bayesian MCMC analysis. Finally, site selection pressure and glycosylation analyses were performed using FUBAR and NetNGlyc/NetOGlyc tools.

**Results:**

Out of 155, 98 and 57 sequences were RSVA and RSVB, respectively. The tMRCA was estimated to be around 2002 and 2005 for RSVA and RSVB, respectively. RSVA sequences formed two NA1 genotype clusters, comprising 95 and three sequences, respectively. RSVB formed three clusters, where 24 and two sequences clustered with BA9 and BA12 genotypes, respectively, while 31 sequences formed a unique cluster. The RSVA and RSVB glycoprotein gene sequences exhibited N- and O- glycosylation and selection pressure at several sites. RSV B exhibited slightly higher (0.042) nucleotide diversity per site (π) as compared to RSVA (0.019).

**Conclusions:**

Our results suggest that RSVA and RSVB strains in Pakistan exhibit distinct genotypic clusters and differ in their estimated tMRCA. Additionally, both genotypes showed glycosylation and selection pressure at specific sites, with RSVB exhibiting higher nucleotide divergence per site (π), indicating its potential to undergo further evolutionary changes and adaptation. Overall, this study provides unique insights into RSV molecular epidemiology. The study may also help improve our understanding of RSV evolutionary changes and the emergence of new genotypes in different regions worldwide and within Pakistan.

## 1. Introduction

Respiratory syncytial virus (RSV) belongs to the genus Orthopneumovirus within the family Pneumoviridae (Collins et al., 2013; Raffaldi and Castagno, 2024). RSV is a significant cause of respiratory illnesses in children worldwide (Breiman et al., 2013; Hall et al., 2009), responsible for approximately 30 million new acute lower respiratory infections (ALRI) and nearly 199,000 deaths in children less than five years worldwide (Dapat et al., 2010; Do et al., 2012; Aamir et al., 2013; Ahmed and Lyles, 1998); however, the disease burden of RSV in low-and-middle-income countries (LMICs) is less studied (Nair et al., 2010). Worldwide, it is estimated that 33 million cases of ALRI per year in children under five years of age are attribute to RSV; 3.2 million of which require hospitalization, resulting in a substantial increase in the burden of RSV (Shi et al., 2017a; Iwane et al., 2013; Obando-Pacheco et al., 2018), making RSV the most significant contributor to childhood respiratory illness in LMIC (Weber et al., 1998).

RSV can be classified into two antigenic subgroups, A and B, based on the antigenicity of the G protein. The extracellular domain of the G protein gene, which has two hypervariable regions, HVR1 and HVR2, is the most variable region (Zlateva et al., 2007; Moura et al., 2003). G gene C-terminal hypervariable region has been used to further divide RSV A and B into genotypes, where RSV-A has been classified into 22 genotypes, namely GA1–7, SAA1–2, NA1–4, ON1–4, CB-A, TN1–2, and LBA1–2. In comparison, RSV-B has been classified into 36 genotypes, namely GB1–13, THB, BA1–14, BA-CCA, BA-CCB, SAB1–4, and URU1–2. Genetic variability in the G gene is the leading cause of the emergence of new genotypes. In contrast to other respiratory viruses, such as influenza, RSV has diverse circulation patterns, with the presence of cocirculating genotypes within the same epidemic region (Tsukagoshi et al., 2013).

Due to various causes, Pakistan has one of the highest rates of child death globally, yet little is known about the contribution RSV plays in child mortality (Tsukagoshi et al., 2013; Liu et al., n.d.; Williams et al., 2002). To date, only three studies have been published on the molecular epidemiology of RSV in the Northern parts of Pakistan. However, the phylodynamic changes associated with the RSV epidemic in these regions have still not been studied. Karachi is the largest urban city in Pakistan, with a high burden (19 %) of RSV among children presenting with respiratory illness. Despite this, there is limited data on the molecular epidemiology and phylogenetics of RSV and no data on the phylodynamic characterization of RSV (Ali et al., 2017a).

This study aimed to perform genetic, phylogenetic, and phylodynamic analyses of RSV strains circulating in children under five years old in Karachi, Pakistan.

## 2. Material and methods

### 2.1. Study design, samples, RNA extraction, cDNA synthesis, RSV glycoprotein (G) gene amplification and sequencing

This was a retrospective, cross-sectional study. Previously, nasopharyngeal swab samples from 1150 children less than five years of age were recruited during 2009–2012 and tested for RSV and other respiratory pathogens, including influenza A, B, H1N1, and human metapneumovirus, using RT-PCR, after obtaining written informed consent from the parents or guardians (Ali et al., 2017a). The inclusion criteria for enrolment in this study included being under 5 years of age and presenting with any of the following symptoms: ARIs, apnea, asthma exacerbation, bronchiolitis, croup, febrile seizure, respiratory distress, otitis media, pharyngitis, pneumonia, sinusitis, and upper respiratory infection (URI). This study was approved by the Ethics Review Committee (1239-Ped-ERC-09 and 2023–9282-26,469) of Aga Khan University, Karachi, Pakistan.

Nasopharyngeal (NP) swabs were obtained using commercial flocked swabs (Diagnostics Hybrid Inc.) and were immediately transported to the Aga Khan University Hospital on ice to maintain a cold chain of 2–8 °C for further PCR testing. Out of 1150, 223 samples were found positive for RSV on real-time RT-PCR in the previous study (Ali et al., 2017a). In the current study, out of 223, 155 samples with a Ct-value of ≤30 were used to perform RNA extraction using a commercially available QIAmp viral RNA mini kit (QIAGEN, Hilden, Germany) following the manufacturer’s instructions. The RNA samples were stored at −80 °C until further use.

The G gene of RSV was amplified using a two-step nested PCR strategy. Reverse transcription and first-round amplification were performed using a QIAGEN One-Step RT-PCR kit. The first-round primers were AG20OF: 5′-GGGGCAAATGCAAACATGTCC-3′; F164OR: 5′-GTTATGACACTGGTATACCAACC-3′, while the second-round primers were BG10IF: 5′ GCAATGATAATCTCAACCTC-3′ and F1: 5′-CAACTCCATTGTTATTTGCC-3′. In each round, 0.4 pM/μL of primers were used. The thermocycling conditions were as follows: 50 °C for 30 min, 95 °C for 15 min, followed by 40 cycles of 94 °C for 30 s, 54 °C for 30 s, and 72 °C for 1 min with the final extension of 10 min at 72 °C. One microlitre of the first-round PCR product was used for the second-round PCR. The thermocycling conditions were as follows: 95 °C for 2 min, followed by 30 cycles of 95 °C for 45 s, 54 °C for 45 s, and 72 °C for 1 min, followed by a final extension at 72 °C for 5 min (Agoti et al., 2012). The amplified products were visualized on a 2 % agarose gel (Sigma-Aldrich A1296; St. Louis, MO, USA). Additionally, genotype-specific primers were used to sequence. For genotype A, G533 forward primer (5′-TGTAGTATATGTGGCAACAA-3′) and for genotype B forward primer (5′-TGTAGTATATGTGGCAACAA-3′) was used. PCR products were sequenced by Macrogen Inc., Korea. The sequence data obtained from this study was deposited to GenBank, and the following accession numbers were assigned: OK078630-OK078727 and OK078728-OK078784.

### 2.2. Genotyping and phylogenetic clustering analysis

For genotyping, RSV G gene sequences were individually analyzed using the BLAST tool available on the NCBI database, and each sequence was assigned an A and B genotype based on the BLAST results. In the next step, RSVA (*n* = 70) and RSVB (*n* = 45) reference sequences were retrieved after BLAST search and aligned with our study RSVA (*n* = 98) and RSVB (*n* = 57) G gene sequences, separately, using the Mega X software (Kumar et al., 2018). The alignments were subsequently used to construct Maximum likelihood (ML) trees using the PhyML tool available at the HIV Los Alamos sequence database (Guindon et al., 2010), using the following parameters: substitution model – HKY85; test of phylogeny –Shimodaira-Hasegawa approximate likelihood-ratio test (SH-aLRT) branch support and bootstrap of 1000 replicates. The ML tree was visualized and colored using Figtree v1.3.1 software (Lemey et al., 2009). The phylogenetic clusters were identified using an approximate Likelihood Ratio Test (aLRT) with a node support value threshold of ≥90 %, which provides strong statistical support for the branching patterns in the phylogenetic tree.

### 2.3. Time-scaled phylogenetic reconstruction and evolutionary rates

The RSVA and RSVB G gene sequences were separately used to carry out Bayesian time-scaled phylogenetic reconstruction. Both RSVA and RSVB sequence datasets were divided based on the clusters identified in initial phylogenetic analysis; for RSVA, the sequences were divided into a) all RSVA gene sequences, b) NA1 cluster 1, and c) NA1 cluster 2, while for RSVB, the sequences were divided into a) all RSVB gene sequences, b) BA9, c) a unique cluster, and d) BA12 cluster. Since our study sequences (*n* = 98 RSVA, and *n* = 57 RSVB) were collected during 2009–2015 and did not independently exhibit a sufficient temporal signal for inference of the dates of origin, additional Pakistani reference sequences (*n* = 70 RSVA, and *n* = 45 RSVB) matching the study sequences in NCBI BLAST search, were combined to inform the temporal signal.

The time to the most common recent ancestor (tMRCA) and effective population size of RSV genotypes A and B were estimated using a Bayesian Markov Chain Monte Carlo (MCMC) analysis in BEAST software v1.10 (Drummond and Rambaut, 2007). Evolutionary rates and dated trees were estimated using a Bayesian Markov Chain Monte Carlo (MCMC) technique on Beast v. 1.7.4 with HKY model parameters and relaxed lognormal molecular clocks. A Bayesian skyline model with 100 × 10^8^ generations was constructed. Convergence was accepted based on an effective sampling size (ESS) value of >200. The 95 % highest posterior density (HPD) intervals were used to estimate times to the most recent common ancestor (tMRCA) certainty. Using the Tree Annotator program, the maximum clade credibility tree (MCC) was constructed after discarding 10 % of sequences as burn-in.

### 2.4. d_N_/d_S_ and recombination analysis

To evaluate the evolutionary divergence and selection pressure on RSVA and RSVB G gene sequences, the number of synonymous (d_S_) and non-synonymous (d_N_) nucleotide substitutions were estimated using FUBAR analysis available at Datamonkey server (Murrell et al., 2013). Similarly, to identify any recombinant strains in our RSVA and RSVB datasets, the GARD analysis was performed using the Datamonkey webserver (Pond et al., 2007).

### 2.5. N- and O-glycosylation site analysis

Putative N-glycosylation (Asn-X-Ser/Thr) and O-glycosylation sites in RSVA and RSVB glycoprotein amino acid sequences were predicted using NetNGlyc 1.0 (Gupta and Brunak, 2002) and NetOGlyc 3.1 (Chen et al., 2008) webservers, respectively.

### 2.6. Genetic diversity analysis

The mean genetic nucleotide diversity (π) and differentiation coefficient between RSVA and RSVB subpopulations were calculated using MEGA7 software (Nei and Kumar, 2000; Kumar et al., 2016). Analyses were conducted using the Kimura 2-parameter model (Kimura, 1980).

## 3. Results

### 3.1. Patient’s profile and seasonal variability in RSV infection

The clinical and demographic data for 155 samples shortlisted for this study showed that 65 % of the enrolled children were male. The most common diagnosis in RSV-positive children was bronchiolitis (53 % and 56.5 % in hospital and community samples), followed by pneumonia (Table 1).

**Fig. 1.**
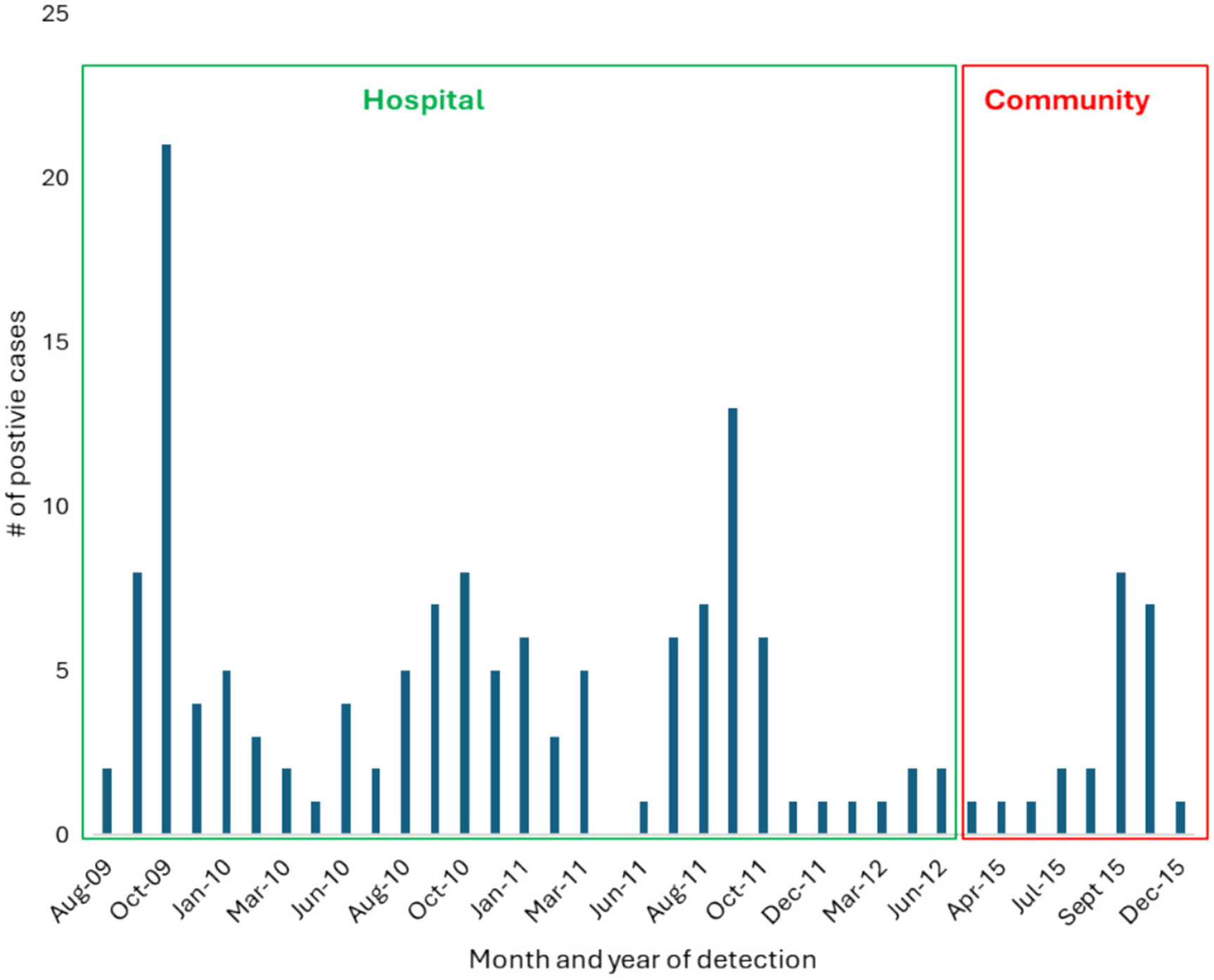
Seasonal distribution of RSV infections in children with acute ARI in Karachi, Pakistan, from 2009 to 2015. The bars represent the number of RSV-positive cases (Y-axis) observed during different months from 2009 to 2015 (X-axis).

The seasonality analysis showed that RSV cases were detected year-round with a 1.6–46 % frequency; however, the RSV peaks were observed from August to October, with a substantial increase in September (Fig. 1).

### 3.2. RSV genotype distribution and phylogenetic cluster analysis

**Fig. 2.**
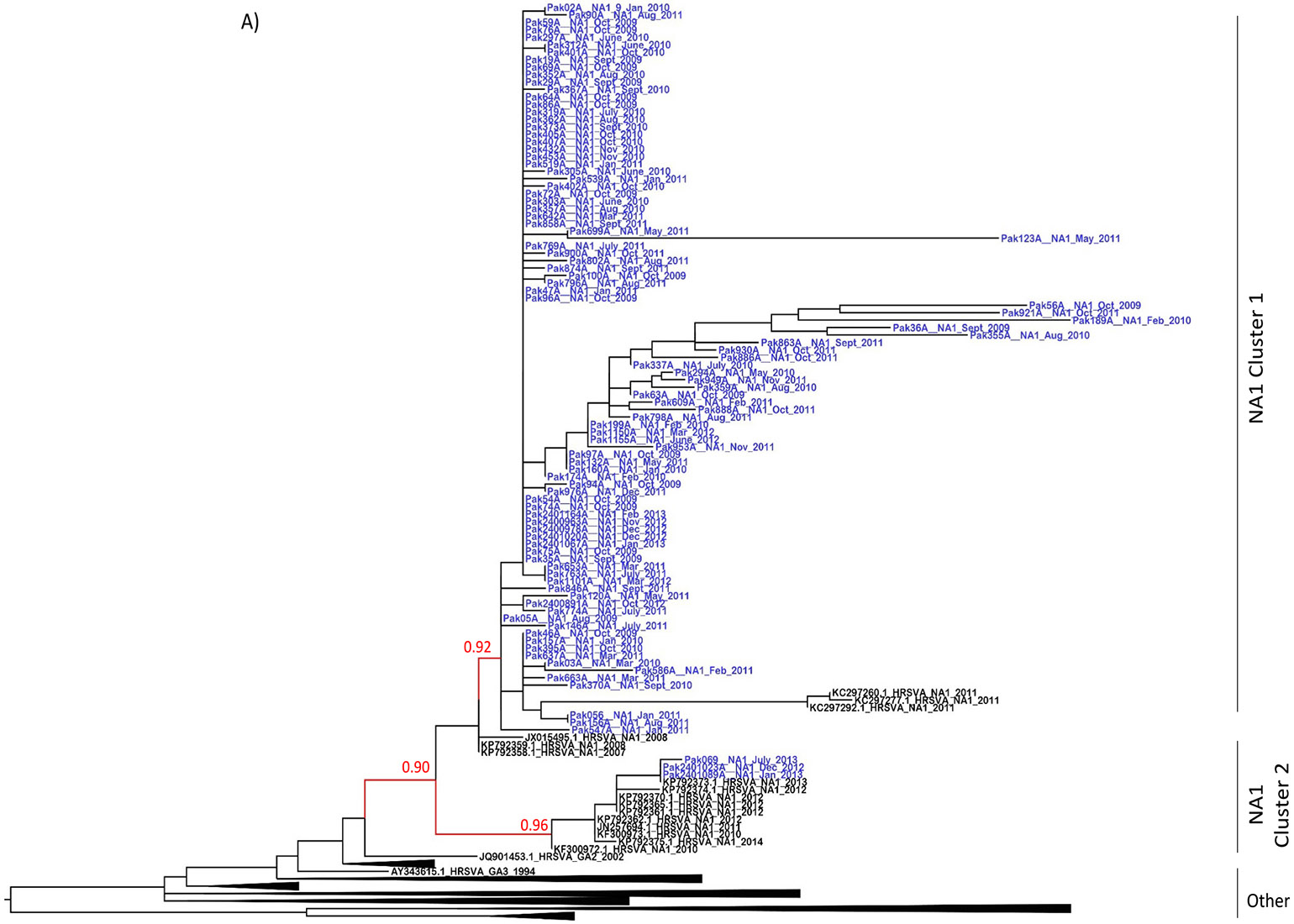

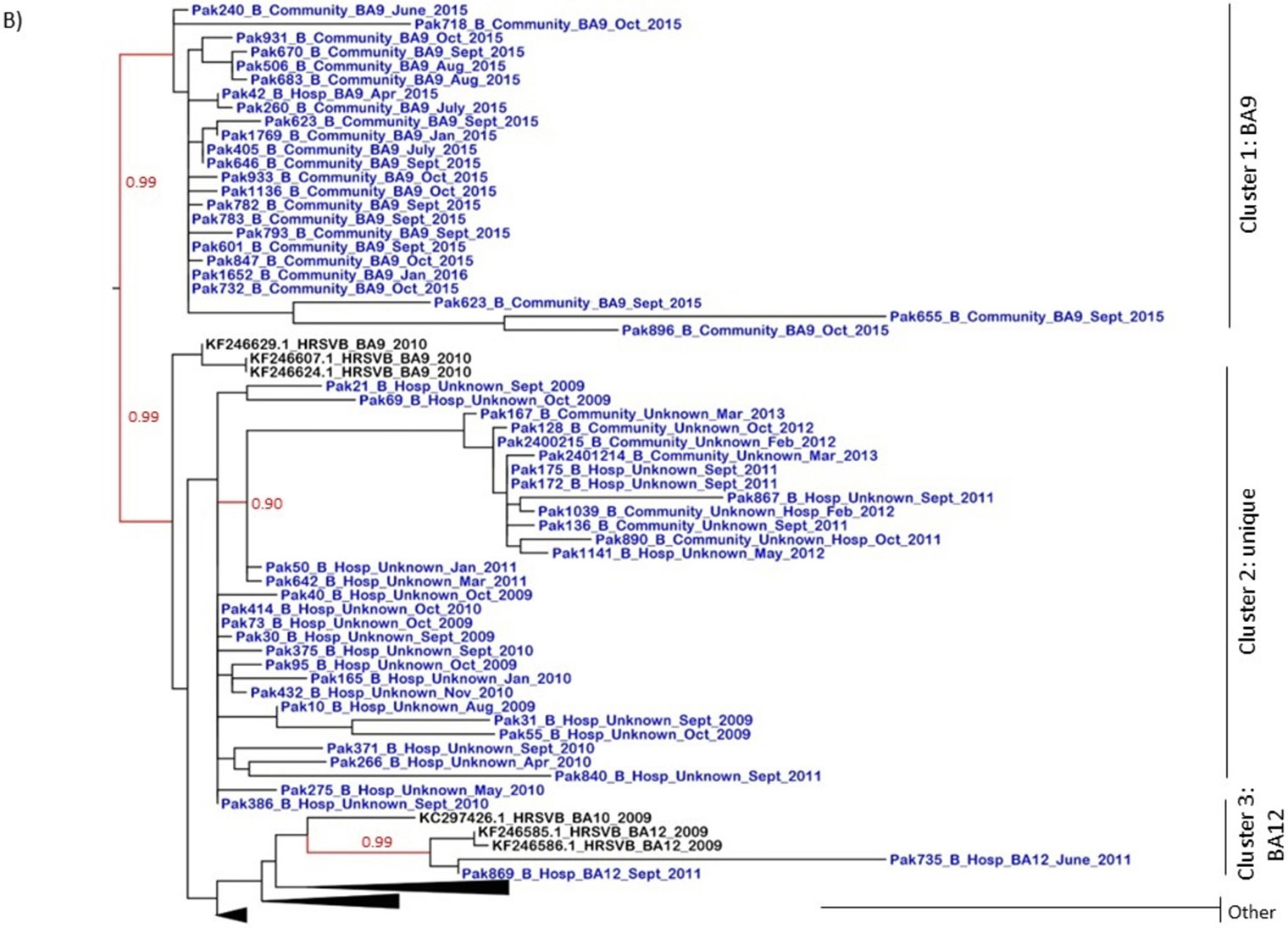
Maximum likelihood (ML) tree of RSV G gene sequences. Maximum likelihood tree for A) RSVA and B) RSVB gene sequences. The study sequences are represented in blue, whereas black represents reference sequences. The tree scale represents the nucleotide per substitution site. Nodes with significant (≥90) aLRT values are shown in red colour.

The BLAST analysis revealed that out of 155, 98 and 57 sequences were RSVA and RSVB, respectively. The ML phylogenetic analysis of RSVA Pakistani sequences showed that RSVA sequences formed two clusters matching the NA1 genotype. In cluster 1, 95 sequences formed a separate NA1 cluster (SH-aLRT node support: 92), whereas in cluster 2, the remaining 3 RSVA gene sequences clustered with NA1 genotype reference sequences (SH-aLRT node support: 96) (Fig. 2A). On the contrary, RSVB Pakistani sequences formed three different clusters: in cluster 1, 24 RSVB sequences formed a cluster with BA9 sub-genotype (SH-aLRT node support: 99); in cluster 2, 31 RSVB sequences formed a unique cluster (SH-aLRT node support: 90) which was distinct from all known RSVB genotype sequences; and in cluster 3, the remaining 2 RSVB sequences clustered with BA12 reference sequences (SH-aLRT node support: 99) (Fig. 2B).

**Fig. 3.**
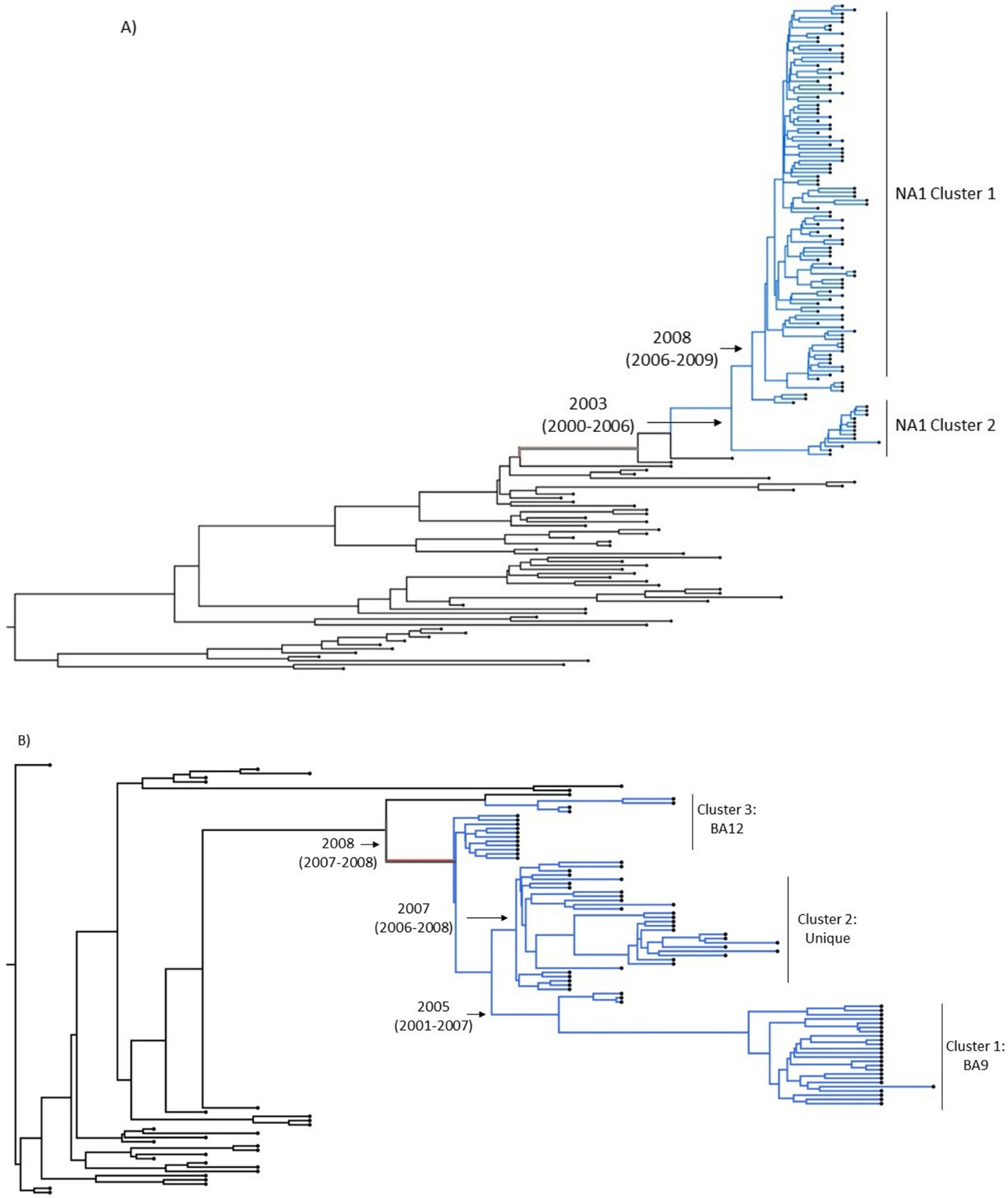
Maximum clade credibility tree of RSV G gene sequences. MCC tree for RSVA (A) and RSVB (B) gene sequences. The tree scale represents the nucleotide per substitution site. Nodes in black colour represent reference sequences exhibiting no clusters. A) The blue colour represents the study sequences forming a cluster around NA1 cluster 1 with a node age of 2008 and an NA1 cluster 2 with a node age of 2003. B) Blue nodes represent the study sequences forming a cluster with the BA9 genotype with a node age of 2005, a unique cluster with a node age of 2007, and a third cluster with a BA12 genotype with a node age of 2008.

The clustering patterns observed on the ML were corroborated by the Bayesian MCC tree, which showed similar clustering patterns for Pakistani RSVA (Fig. 3A) and RSVB (Fig. 3B) genotypes, respectively. Additionally, the MCC tree showed a node age of NA1 cluster 1 to be 2008 and NA1 cluster 2 to be 2003 (Fig. 3A). Similarly, the node age of the cluster BA9 was 2005; for the unique cluster, 2007; and for BA12, it was 2008 (Fig. 3B).

### 3.3. Time to the most common recent ancestor (tMRCA), effective population size, and evolutionary rate estimation

**Fig. 4.**
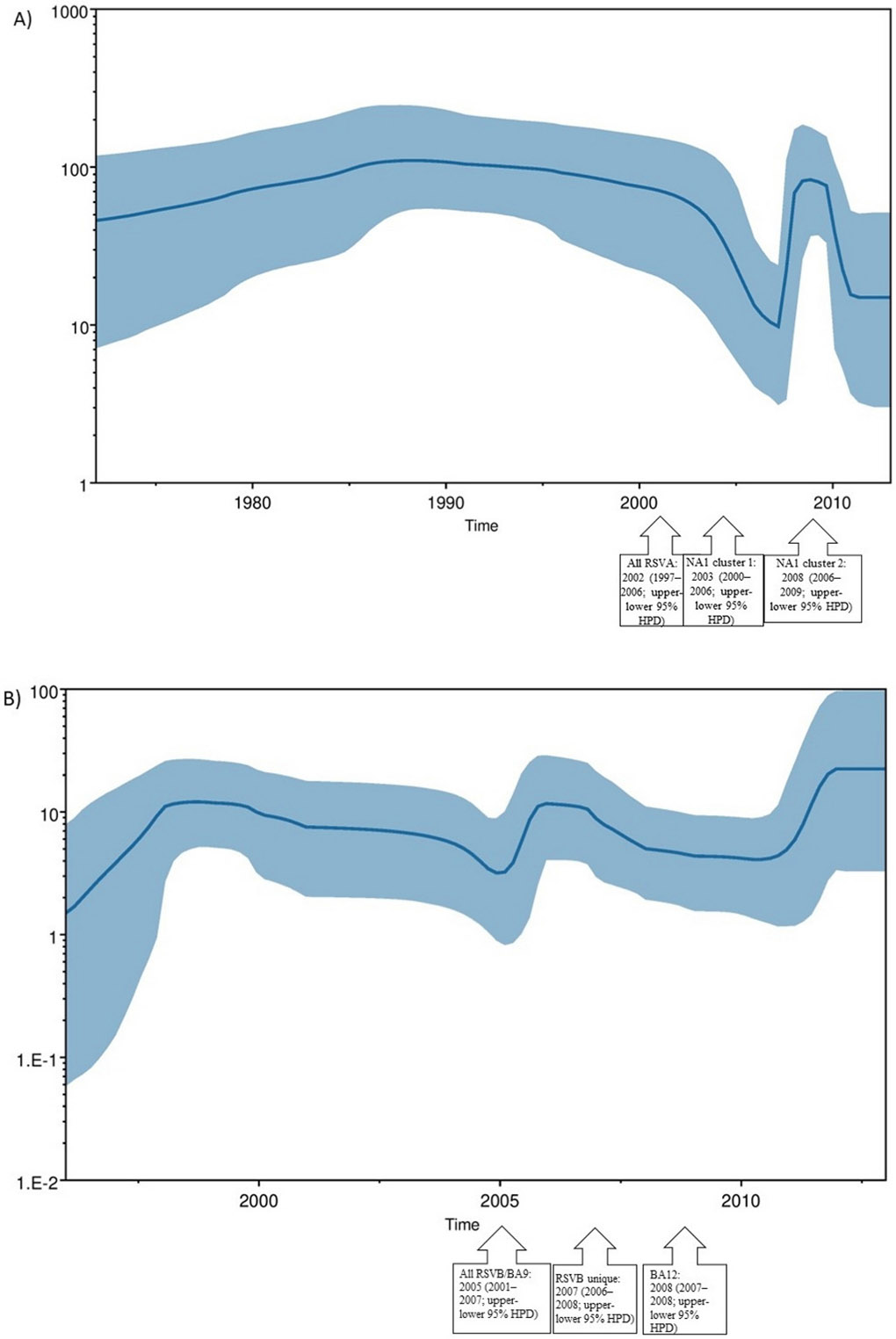
Bayesian skyline plot for Pakistani RSV sample and reference G gene sequences. The plot is shown for A) RSVA and B) RSVB Pakistani RSV sequences. The Y-axis shows effective population size, while the time in years is shown on the X-axis. Blue areas represent a 95 % HPD interval, whereas the dotted line shows the tMRCA. Arrow callouts show tMRCA for clusters observed for RSVA and RSVB sequences.

The tMRCA was estimated to be around 2002 (1997–2006; upper-lower 95 % HPD) for all RSVA sequences, while for NA1 cluster 1 and cluster 2, tMRCA was around 2008 (2006–2009; upper-lower 95 % HPD) and 2003 (2000–2006; upper-lower 95 % HPD), respectively (Fig. 4A, black-boxed arrows).

Similarly, the tMRCA for all RSVB sequences was estimated to be 2005 (2002–2007; upper-lower 95 % HPD), for the BA9 cluster 2005 (2001–2007; upper-lower 95 % HPD), for the unique cluster 2007 (2006–2008; upper-lower 95 % HPD), and for the BA12 cluster 2008 (2007–2008; upper-lower 95 % HPD) (Fig. 4B, black-boxed arrows).

The evolutionary rates for RSVA and RSVB G gene sequences were estimated to be 2.36 × 10^−3^ and 1.03 × 10^−2^ substitution/sites/year, respectively. Moreover, the past population dynamics of RSVA exhibited a pattern of steady expansion followed by a decline, succeeded by a sudden surge during the decade spanning from 2000 to 2010 (Fig. 4A). Similarly, for RSVB gene sequences, the effective population size displayed a continuous growth and decline throughout the decade from 2000 to 2010, followed by a consistent expansion from 2012 and continuing thereafter (Fig. 4B).

### 3.4. d_N_-d_S_ analysis

**Fig. 5.**
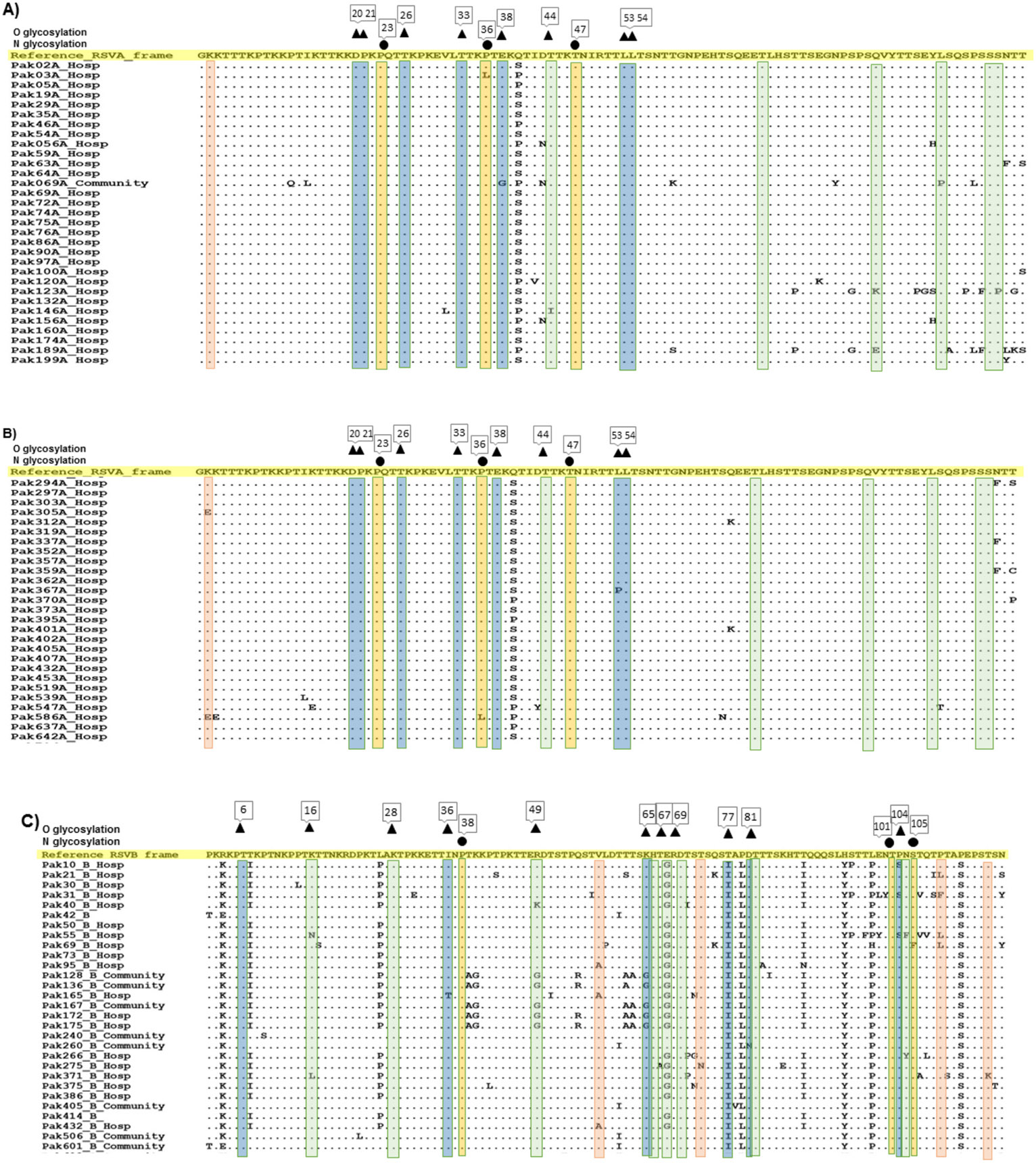

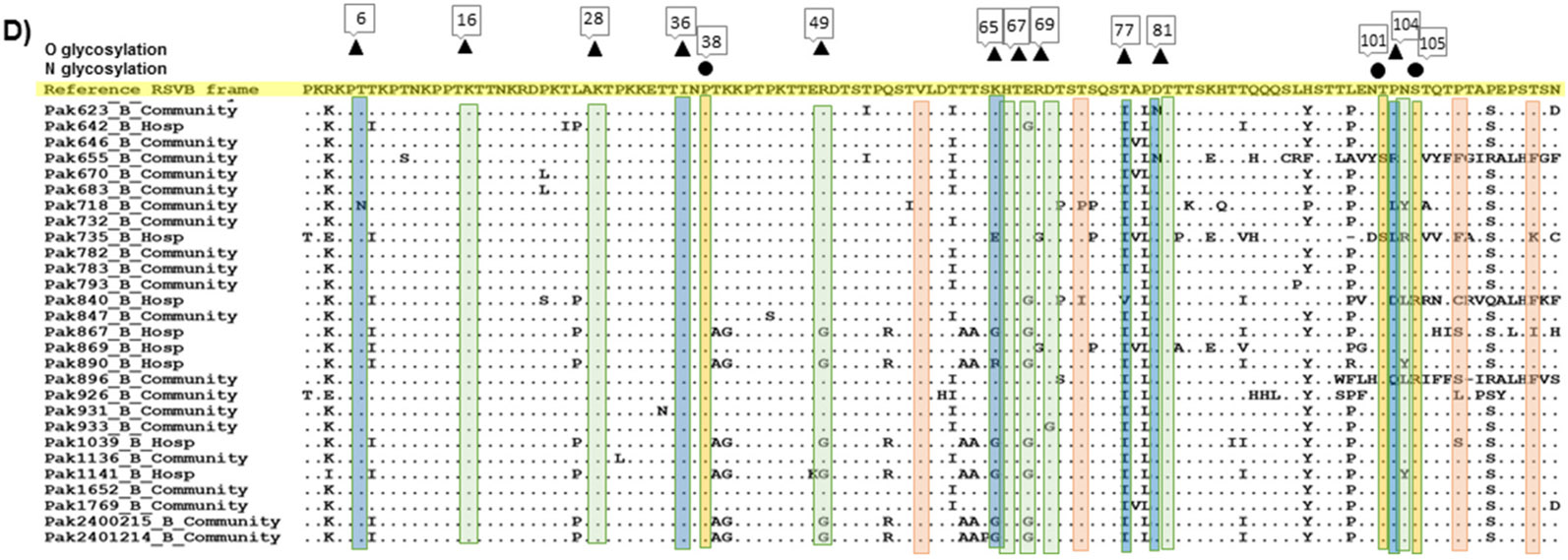
Amino acid alignment of RSV G amino acid sequences with O and N glycosylated and negative and positive selection sites. Amino acid alignment of RSVA (A and B) and RSVB (C and D) Pakistani sequences. The yellow highlighted sequence represents the reference sequence (NA1 for RSVA and BA9 for RSVB). The blue and orange highlighted sequence with inverted triangle and circle shape represent O and N glycosylated sites, respectively, while the green highlighted sequence denotes negative selection sites, and the red highlighted sequence denotes positive selection sites.

The d_N_-d_S_ analysis showed that the overall mean_dN-dS_ ± SE (standard error) value for RSVA G gene sequences was −1.0 ± 0.388, where one site (at position 2) was under positive selection pressure. In contrast, five sites (at positions 45, 71, 85, 98, and 99) were under negative selection pressures (Fig. 5A). Similarly, for RSVB gene sequences, the overall mean_dN-dS_ ± SE was estimated to be −0.5 ± 0.20. Four sites (at positions 58, 72, 107, and 118) were found to be under positive selection pressure, while eight sites (at positions 16, 28, 49, 65, 67, 69, 80, and 102) were under negative selection pressures (Fig. 5B).

### 3.5. N- and O-glycosylation sites

The analysis of putative O-glycosylation sites in all RSVA gene sequences showed eight O-glycosylated sites with a G-score of 0.2–0.39 at positions 20, 21, 26, 33, 38, 44, 53, and 54 (Fig. 5A and B). Analysis of 95 sequences that formed the NA1 cluster 1 showed that one sequence had three N-glycosylated sites at positions 23, 36, and 47. The three RSVA sequences within NA1 cluster 2 showed no glycosylation site/sequence.

Analysis of all RSVB G gene sequences, including those from clusters BA9 and BA12, revealed potential O-glycosylation sites with G-scores ranging from 0.49 to 0.79 at positions 6, 16, 28, 36, 49, 65, 67, 69, 77, 81, and 104 (Fig. 5C and D). Additionally, N-glycosylation sites were identified at positions 38, 101, and 105 in all RSVB gene sequences and the sequences from clusters BA9 and BA12.

### 3.6. Diversity analysis

Population nucleotide diversity was calculated between subpopulations of RSVA and RSVB based on their phylogenetic clusters. The total nucleotide diversity per site (π) for RSVA was 0.019, with a coefficient difference of −0.039. Similarly, the total nucleotide diversity per site (π) for RSVB was 0.042, with a coefficient difference of 0.335 within subpopulations. The analysis revealed that Pakistani RSVB strains are more genetically diverse and structured into distinct subpopulations compared to RSVA, which showed lower diversity and less differentiation among its clusters.

## 4. Discussion

Worldwide, acute lower respiratory infection (ALRI) is a significant cause of morbidity and mortality in children younger than five years, with RSV being identified as the most common viral pathogen responsible for ALRI in children (Shi et al., 2017a). Pakistan is among those countries with the highest rates of child death globally, yet the contribution RSV plays to child mortality is largely unknown (Tsukagoshi et al., 2013; Liu et al., n.d.; Williams et al., 2002). A handful of studies published on RSV from Pakistan suggest RSV plays a significant role in child mortality and morbidity (Stein et al., 2017; Scheltema et al., 2017). However, the comprehensive molecular epidemiology of RSV, such as strain diversity, phylogenetic relationship among strains, and phylodynamic changes associated with the RSV epidemic in different regions, has still not been performed. Karachi, a major city in Pakistan, has a high burden (19 %) of RSV among children presenting with respiratory illness. Despite this, the molecular epidemiology of RSV in this region is poorly understood (Ali et al., 2017a). In the current study, we have performed a comprehensive genetic and phylodynamic analysis of 155 RSV-positive samples obtained previously from children under five years from community settings and admitted to a tertiary care hospital in Karachi, Pakistan (Ali et al., 2017a).

The clinical and demographic data associated with these samples showed that 65 % of the enrolled children were male. This observation is consistent with previously published data that of all RSV-positive children, male children have a higher (55 %) proportion of RSV as compared to females (Stein et al., 2017; Scheltema et al., 2017). Additionally, the immuno-modulatory effects of male sex hormones during infancy may also play a role in making male infants more susceptible to RSV infection. Furthermore, other significant factors, such as low birth weight, crowded environment, malnutrition, etc., may also contribute to increased susceptibility to RSV infection (Nagayama et al., 2006). Similarly, pneumonia and bronchiolitis were predominant clinical features in RSV-positive cases with ARI. Our findings are also consistent with previously reported data showing 36 % of confirmed pneumonia patients to be RSV-positive (Ali et al., 2013). Another study conducted in Gilgit Baltistan, a northern area of Pakistan, reported that RSV accounts for nearly 85 % of bronchiolitis cases and 20 % of childhood pneumonia cases (Aamir et al., 2013).

RSV seasonality analysis revealed that RSV infections occurred throughout the year. However, the peak of incidence was during July and September each year. Previous studies conducted in India, Thailand, and Malaysia also showed a similar seasonality pattern of RSV infections (John et al., 1991; Naorat et al., 2013; Khor et al., 2012). Our observation is, however, in contrast to the two previously published reports from the Northern region of Pakistan, where RSV infections were frequently detected during the winter season, starting from February, and gradually declined towards the end of March (Aamir et al., 2013; Tariq et al., 2005). This can be due to differences in climatic and environmental factors, such as temperature, pollution, etc., between the Northern and Southern areas of Pakistan. Previous studies have shown that RSV outbreaks occur during winter months in Mediterranean climates, whereas in tropical regions, the peak of RSV infection is usually seen after seasonal rainfalls (Weber et al., 1998). RSV outbreaks in tropical areas mainly occur during the wet seasons following the onset of seasonal rainfall. Countries near the equator with consistently high rainfall exhibit a noticeable trend of RSV that occurs throughout the year (Divarathna et al., 2022; Ali et al., 2017b). RSV shows a seasonal infection pattern based on the population and geography. Some evidence suggests that certain climate factors, for example, in European countries, temperate climates with low temperatures and increased relative humidity, are associated with RSV infections (Radhakrishnan et al., 2020). On the other hand, in tropical settings, RSV infections typically peak after seasonal rainfalls (Ali et al., 2017b; Yassine et al., 2020).

The genetic analysis of RSV sequences showed the presence of both RSV genotypes A and B genotypes. However, the prevalence of RSVA was higher (63.2 %) than RSVB. These findings are different from previous reports published from Northern Pakistan, where RSV B was found to be the dominant genotype (Bashir et al., 2017a), suggesting differences in genotype distributions between different regions of Pakistan (Cantú-Flores et al., 2022). A study published in 2022 analyzed the worldwide distribution of RSV A and B, using sequences from GenBank, and revealed RSV A to be responsible for most of the reported cases. The plausible explanation is the combination of geographical locations and the high population of humans living north of the equator. Another factor is the limited data available from countries located in the southern region. After 2000, the majority of studies focused on the prevalence and seasonality of RSV B compared to RSV A. However, the limited data is available from Asia (Stein et al., 2017).

The ML phylogenetic analysis of these RSVA sequences from Pakistan showed that they clustered into two distinct groups corresponding to the NA1 genotype. Specifically, 61.2 % of sequences formed one NA1 cluster (cluster 1), while 1.9 % of sequences formed a separate NA1 cluster (cluster 2). Data from 2006 onwards have reported RSVA NA1 strains to be present in different populations worldwide (Shobugawa et al., 2009; Arnott et al., 2011; Peret et al., 2000; Baek et al., 2012). Previous studies from Pakistan also reported RSV A NA1 strain to be the only genotype largely present in collected samples (Aamir et al., 2020). The Bayesian MCC tree analysis also reaffirmed this clustering pattern and provided insights into the temporal aspects of these clusters.

Phylodynamic analysis of RSV A NA1 strain showed the node age of cluster 1 and cluster 2 to be 2008 and 2003, respectively, while the time to the most recent common ancestor (tMRCA) for all RSVA gene sequences was estimated to be around 2002. The mean evolutionary rate of RSV A was estimated to be 2.36 × 10^−3^, which was close to the rates reported by previous studies, including the Philippines (5.40 × 10^−3^), Italy (2.10 × 10^−3^) and Japan (4.61 × 10^−3^) (Malasao et al., 2015; Martinelli et al., 2014; Hirano et al., 2014). The population dynamics analysis of RSVA exhibited a pattern of steady growth, subsequent decline, and a sudden resurgence during the decade spanning from 2000 to 2010. This pattern could reflect changes in host population immunity, viral transmission dynamics, or other epidemiological factors influencing the virus’s spread and evolution.

The average dN/dS ratios for RSV A indicated mixed selective pressures, revealing two distinct sets. One site (position 2) was under positive selection, while five sites (positions 45, 71, 85, 98, and 99) were under negative selection pressures. The positive dN-dS analysis revealed evidence suggesting that while most of the gene is conserved, certain positions could play a critical role in the virus’s fitness and are consequently subjected to selective pressures. The dN-dS ± SE of −1.0 ± 0.388 indicates that the RSVA population has recently expanded or undergone positive selection, as reported previously (Shishir et al., 2023a). It has been reported that N and O glycosylation helps the virus in evading immune response (Cane et al., 1991; Rajala et al., 2003). NA1 cluster 1 exhibited three putative sites, which may contribute to antigenic variability, whereas none was observed in cluster 2. These differences in glycosylation patterns could have implications for the virus’s antigenicity and its interaction with the host immune system (Parveen et al., 2006). The changes in glycosylation patterns, especially the loss or gain of O-glycosylation sites, can affect how well the virus evades the host immune system. For instance, gaining new O-glycosylation sites might help the virus avoid immune detection, while the loss of such sites could potentially make the virus more susceptible to immune responses (Gardinassi et al., 2012). For example, some RSV strains that had O-glycosylation sites at positions 296 and 297 showed less severe disease symptoms, while strains with O-glycosylation site at position 293 were more pathogenic and with more severe disease outcomes (Gardinassi et al., 2012). Also, Glycosylation patterns and amino acid substitutions in RSV are pivotal in shaping the virus’s evolution and immune evasion. For example, a study from Taiwan showed that N-glycosylated sited at 274 in ON1 and 274 in NA1 were under positive selection, suggesting these glycosylation patterns may be crucial for viral adaptation or immune evasion (Lee et al., 2021).

The ML phylogenetic analysis of RSVB sequences identified three distinct clusters: cluster 1, comprising of BA9 sub-genotype, suggesting a genetic lineage shared with previously characterized strains; cluster 2, comprising BA12 sequences, indicating another distinct genetic lineage present in the sampled population; and cluster 3 formed by Karachi-specific strains, distinct from the BA9, BA12 or any other RSVB BA strains, indicating a local evolution or a novel genetic lineage specific to the region. The RSVB BA strains were first reported in Argentina in 1999 (Trento et al., 2003) and since then have been widespread globally and developed at least 13 different variants to date (Trento et al., 2010; Bashir et al., 2017b). The most observed RSVB sub-genotype in Pakistan is BA9, reported in a mass survey conducted between 2010 and 2013 (Aamir et al., 2020). BA12 strains have been reported in a few countries, such as Rome (Lee et al., 2021) and Malaysia (Trento et al., 2003), while none have been reported in Pakistan, suggesting BA12 to be present in a few parts of the country and may have been introduced via traveling. These clustering patterns were further supported by the Bayesian MCC tree, which confirmed the presence of these clusters and provided estimated node ages.

The node age of BA9 was estimated to have emerged around 2005 in our population, while the BA12 sub-genotype was estimated around 2008, but due to limited data available for this strain, we can presume this to be a travel or migration-associated infection that could have been introduced by travelers coming to Pakistan. Interestingly, the node age of our unique RSVB cluster was estimated to be around 2007, and around the same year, a UNICEF report described a new RSV outbreak among Asian countries (Unicef, 2009), which could provide insights into the evolutionary history and emergence of RSV. Furthermore, we also estimated the evolutionary rates of the RSVB G gene sequences to be 1.03 × 10^−2^ substitution/sites/year. This rate was lower than the previously reported evolutionary rates for the RSVB Glycoprotein gene (Yu et al., 2021). The effective population size analysis of RSVB showed fluctuations from 2000 to 2010, with a notable expansion phase beginning around 2012 and continuing thereafter. These dynamics likely reflect the interplay between viral adaptation, host immunity, and environmental factors influencing viral spread and persistence (Tripp, 2013).

Compared to the RSVA sequences, the RSVB BA9, BA12, and unique strains exhibited similar patterns of relatively low dN/dS ratio (mean dN-dS = −0.5 ± 0.20). The number of positively selected sites (four positions) was less than the negative selection sites (eight positions), as seen in a previous study (Yu et al., 2021). These sites highlight regions of the genes potentially involved in adaptation to host immune responses and viral fitness (Oshansky et al., 2009). Similarly to the selection pressure, all RSVB G gene sequences, including the three clusters, exhibited specific glycosylation sites (e.g., at positions 6, 16, 28, 36, 49, 65, 67, 69, 77, 81, and 104 for O-glycosylation; positions 38, 101, and 105 for N-glycosylation). These sites may reflect the interplay between the virus’s antigenicity and the host immune system (Parveen et al., 2006).

The findings of NA1 genotype clusters in RSVA and BA9/BA12 genotypes in RSVB partly align with global RSV evolutionary patterns. Recent global surveillance data, especially from China, Korea, and Brazil, showed a shift in genotypic diversity, with ON1 and BA (BA4 and BA9 in China and Korea and BA4 in Brazil) becoming the predominant lineages detected for RSV A and B, respectively, since 2014 (Nuttens et al., 2024). Corroborating our observation for RSVB (nucleotide diversity per site (π) of 0.042 and a positive coefficient of 0.335), current global data also indicates that RSV-B demonstrates greater genotypic diversity (with at least 37 genotypes) than RSV-A (Nuttens et al., 2024). The difference in RSV B genetic diversity, especially the emergence of a unique cluster, may be reflective of differences in immune selection pressure (Shi et al., 2017b). Studies have shown that RSVA and RSVB show a comparable mutation density (RSVA: 0.00135 mutations per base, RSVB: 0.00124 mutations per base) and rate; however, RSVB shows a slightly higher rate of missense mutations (RSVA: 35.85 %, RSVB: 38.23 %) (Agoti et al., 2019; Shishir et al., 2023b), which may explain higher mean diversity observed for RSVB strains in our study. Such variation may have an effect on viral evolution as well as on the emergence of newer variants.

We anticipate certain limitations of our study. Firstly, the children were recruited from a single hospital and community setting, which does not portray the entire paediatric population of Pakistan. Secondly, similar to previously published studies, the sequence analysis was focused only on the G gene; including additional genes or the whole genome may provide deeper insights into RSV genetic diversity and evolutionary dynamics.

Nonetheless, our findings provide a comprehensive understanding of the genetic diversity, evolutionary dynamics, and selective pressures acting on the RSVA and RSVB glycoprotein (G) gene sequences in Karachi, Pakistan, and provide unique insights into RSV evolutionary changes and the emergence of new genotypes in different regions worldwide. The findings from this study further emphasize the importance of continued molecular surveillance to identify variant genotypes of RSV circulating in different geographic areas at different time points within high-risk populations. Genomic surveillance can also serve as an effective early warning system for RSV outbreaks in Pakistan. Similar global studies have shown that systematic RSV genomic surveillance can effectively track viral evolution and spread patterns, such as through air travel (Langedijk et al., 2024), which is particularly relevant for a major metropolitan hub such as Karachi. In this regard, the implementation of whole-genome sequencing methods, coupled with phylogenetics and epidemiological data, can especially aid in the early detection and monitoring transmission of specific genotypes and variants of concern (with rare mutations) that may affect viral pathogenicity and vaccine efficacy (Evans et al., 2024).

## Supplementary Material

1

## Figures and Tables

**Table 1:** The demographic characteristics of RSV PCR-positive children.

Baseline characteristics	RSV positive Hospital	RSV positive Community
	*n* = 132	*n* = 23
Median age (in months)	5.5	4.5
0–2 n (%)	31 (23.5)	17 (73.9)
3–5	41 (31.0)	3 (13.0)
6–11	37 (28.0)	2 (8.7)
12–60	23 (17.4)	1 (4.4)
Male n (%)	86 (65.2)	12 (52.2)
Female n (%)	46 (34.9)	11 (47.8)
Clinical Characteristics		
Pneumonia	51 (38.6)	12 (52.2)
Bronchiolitis	70 (53.0)	13(56.5)
Fever	90 (68.2)	20 (86.9)
Cough	120 (90.0)	21 (91.3)
wheezing/noisy breathing	71 (53.8)	16 (69.6)

## Data Availability

All data is available in the manuscript. The sequence data obtained from this study was deposited to GenBank, and the following accession numbers were assigned: OK078630-OK078727 and OK078728-OK078784.
