# Supplementary material for: Phylogenetic and phylodynamic analysis of respiratory syncytial virus strains circulating in children less than five years of age in Karachi-Pakistan
Source: Infect Genet Evol. Author manuscript; Available in PMC 2025 Jan 15. (PMC11733314; doi:10.1016/j.meegid.2024.105694)
Supplement: 1 [file NIHMS2043198-supplement-1.docx]

**Accession numbers with RSV type A and Year of sequence submitted:**

AF065254.1_HRSVA_GA4_1995, AF065255.1_HRSVA_GA5_1995, AF065256.1_HRSVA_GA3_1995, AF065257.1_HRSVA_GA1_1994, AF065407.1_HRSVA_GA1_1988, AF193317.1_HRSVA_GA3_1995, AF193318.1_HRSVA_GA5_1995, AF193319.1_HRSVA_GA3_1995, AF193320.1_HRSVA_GA7_1995, AY114149.1_HRSVA_GA2_1989, AY114150.1_HRSVA_GA5_1980, AY114151.1_HRSVA_GA5_2000, AY343578.1_HRSVA_GA5_2001, AY343586.1_HRSVA_GA5_1993, AY343615.1_HRSVA_GA3_1994, AY343621.1_HRSVA_GA3_1995, AY911262.1_HRSVA_PRO_2005, DQ985122.1_HRSVA_GA3_2005, DQ985123.1_HRSVA_GA3_1997, DQ985131.1_HRSVA_GA6_1998, DQ985132.1_HRSVA_SAA1_1997, HQ731701.1_HRSVA_GA5_1996, HQ731703.1_HRSVA_GA7_1996, HQ731710.1_HRSVA_GA2_1997, JF920069.1_HRSVA_GA1_2007, JN257694.1_HRSVA_NA1_2011, JQ901453.1_HRSVA_GA2_2002, JX015487.1_HRSVA_GA5_2006, JX015495.1_HRSVA_NA1_2008, JX198135.1_HRSVA_GA5_1994, JX513382.1_HRSVA_GA5_2001, KC297260.1_HRSVA_NA1_2011, KC297277.1_HRSVA_NA1_2011, KC297292.1_HRSVA_NA1_2011, KC297324.1_HRSVA_GA3_2011, KC297374.1_HRSVA_GA3_2012, KC297381.1_HRSVA_GA3_2007, KF300972.1_HRSVA_NA1_2010, KF300973.1_HRSVA_NA1_2010, KJ627695.1_HRSVA_GA5_2001, KP258696.1_HRSVA_SAA1_1986, KP258723.1_HRSVA_GA6_1986, KP792358.1_HRSVA_NA1_2007, KP792359.1_HRSVA_NA1_2008, KP792361.1_HRSVA_NA1_2012, KP792362.1_HRSVA_NA1_2012, KP792365.1_HRSVA_NA1_2012, KP792370.1_HRSVA_NA1_2012, KP792373.1_HRSVA_NA1_2013, KP792374.1_HRSVA_NA1_2012, KP792375.1_HRSVA_NA1_2014, KU316099.1_HRSVA_GA1_1997, KU316133.1_HRSVA_GA5_1990, KU316164.1_HRSVA_GA1_1995, M11486.1_HRSVA_GA1_1985, M17212.1_HRSVA_GA1_1987, MG642050.1_HRSVA_GA3_1994, X73351.1_HRSVA_GA7_1989, Z33414.1_HRSVA_GA3_1993, Z33416.1_HRSVA_GA3_1990, Z33417.1_HRSVA_GA7_1992, Z33422.1_HRSVA_GA3_1989, Z33424.1_HRSVA_GA3_1988, Z33426.1_HRSVA_GA3_1990, Z33427.1_HRSVA_GA1_1990, Z33430.1_HRSVA_GA5_1992, Z33431.1_HRSVA_GA1_1991, Z33432.1_HRSVA_GA1_1992, Z33455.1_HRSVA_GA7_1992, Z33494.1_HRSVA_GA5_1990

**Accession numbers with RSV type B and Year of sequence submitted:**

AY333364.1_HRSVB_BA1_1999, AY751105.1_HRSVB_BA6_2001, AY751116.1_HRSVB_BA6_2001, AY751111.1_HRSVB_BA6_2003, AY751117.1_HRSVB_BA6_2002, AY751119.1_HRSVB_BA2_2001, AY751121.1_HRSVB_BA2_2002, AY751122.1_HRSVB_BA2_2003, AY751123.1_HRSVB_BA2_2003, AY751087.1_HRSVB_BA7_2003, DQ171878.1_HRSVB_GB13_2002, DQ227363.1_HRSVB_BA1_1999, DQ227364.1_HRSVB_BA1_1999, DQ227373.1_HRSVB_BA1_2002, DQ227368.1_HRSVB_BA1_2002, DQ227374.1_HRSVB_BA1_2002, DQ227370.1_HRSVB_BA3_2002, DQ227375.1_HRSVB_BA3_2002, DQ227397.1_HRSVB_BA3_2004, DQ227396.1_HRSVB_BA4_2004, DQ227377.1_HRSVB_BA2_2002, DQ227393.1_HRSVB_BA2_2003, DQ227389.1_HRSVB_BA2_2003, DQ227403.1_HRSVB_BA3_2003, DQ227407.1_HRSVB_BA4_2004, DQ227408.1_HRSVB_BA4_2004, KC297426.1_HRSVB_BA10_2009, KF246607.1_HRSVB_BA9_2010, KF246624.1_HRSVB_BA9_2010, KF246629.1_HRSVB_BA9_2010, KF246585.1_HRSVB_BA12_2009, KF246586.1_HRSVB_BA12_2009, KF300952.2_HRSVB_BA14_2008, KF300955.2_HRSVB_BA14_2008, KF300970.2_HRSVB_BA14_2008, KF300953.2_HRSVB_BA14_2008, KF300954.2_HRSVB_BA14_2008, KF300957.2_HRSVB_BA14_2008, KF300958.2_HRSVB_BA14_2008, KF300959.2_HRSVB_BA14_2008, KF300960.2_HRSVB_BA14_2008, KX371868.1_HRSVB_BA14_2008, KX371867.1_HRSVB_BA14_2008, KC297456.1_HRSVB_BAC_2010, KC297486.1_HRSVB_BAC_2009
